# Nanocrystals of Fusidic Acid for Dual Enhancement of Dermal Delivery and Antibacterial Activity: In Vitro, Ex Vivo and In Vivo Evaluation

**DOI:** 10.3390/pharmaceutics12030199

**Published:** 2020-02-25

**Authors:** Iman S. Ahmed, Osama S. Elnahas, Nouran H. Assar, Amany M. Gad, Rania El Hosary

**Affiliations:** 1 Department of Pharmaceutics & Pharmaceutical Technology, College of Pharmacy, University of Sharjah, Sharjah 27272, UAE; 2 Department of Pharmaceutics and Industrial Pharmacy, Faculty of Pharmacy, October 6 University, Giza 12585, Egypt; Osamaelnahas@o6u.edu.eg; 3 Department of Microbiology, National Organization for Drug Control and Research, Cairo 12553, Egypt; 4 Department of Pharmacology, National Organization for Drug Control and Research, Cairo 12553, Egypt; 5 Department of Pharmaceutics, National Organization for Drug Control and Research, Cairo 12553, Egypt; hmrs.loay@gmail.com

**Keywords:** fusidic acid, nanocrystals, lyophilization, ex-vivo studies, rat excision wound infection model, antibacterial activity, dermal drug delivery

## Abstract

With the alarming rise in incidence of antibiotic-resistant bacteria and the scarcity of newly developed antibiotics, it is imperative that we design more effective formulations for already marketed antimicrobial agents. Fusidic acid (FA), one of the most widely used antibiotics in the topical treatment of several skin and eye infections, suffers from poor water-solubility, sub-optimal therapeutic efficacy, and a significant rise in FA-resistant *Staphylococcus aureus* (FRSA). In this work, the physico-chemical characteristics of FA were modified by nanocrystallization and lyophilization to improve its therapeutic efficacy through the dermal route. FA-nanocrystals (NC) were prepared using a modified nanoprecipitation technique and the influence of several formulation/process variables on the prepared FA-NC characteristics were optimized using full factorial statistical design. The optimized FA-NC formulation was evaluated before and after lyophilization by several in-vitro, ex-vivo, and microbiological tests. Furthermore, the lyophilized FA-NC formulation was incorporated into a cream product and its topical antibacterial efficacy was assessed in vivo using a rat excision wound infection model. Surface morphology of optimized FA-NC showed spherical particles with a mean particle size of 115 nm, span value of 1.6 and zeta potential of −11.6 mV. Differential scanning calorimetry and powder X-ray diffractometry confirmed the crystallinity of FA following nanocrystallization and lyophilization. In-vitro results showed a 10-fold increase in the saturation solubility of FA-NC while ex-vivo skin permeation studies showed a 2-fold increase in FA dermal deposition from FA-NC compared to coarse FA. Microbiological studies revealed a 4-fofd decrease in the MIC against *S. aureus* and *S. epidermidis* from FA-NC cream compared to commercial Fucidin cream. In-vivo results showed that FA-NC cream improved FA distribution and enhanced bacterial exposure in the infected wound, resulting in increased therapeutic efficacy when compared to coarse FA marketed as Fucidin cream.

## 1. Introduction

Topical drug delivery is generally recognized as the first-line therapy for skin conditions. Topical antibiotics specifically are preferred to systemic ones in skin infections due to high localization of an antimicrobial agent at the site of infection, thus resulting in reduced risk of systemic toxicity. Topical formulations are also known to be associated with the highest patient compliance because of their easy use [1]. Fusidic acid (FA) is one of the most widely used antibiotics in the treatment of several skin and eye infections to the extent that over 1 million items of FA were prescribed for skin infections in England in 2017. FA is commercially available in the form of cream, ointment, or eye drops. However, the clinical use of topical FA may result in suboptimal therapeutic efficacy due to the pharmaceutical limitations associated with its low water-solubility and poor skin penetration in the prevailing infectious areas from conventional topical dosage forms [2,3]. Moreover, FA-resistant *Staphylococcus aureus* (FRSA) within hospital inpatients, non-dermatology outpatients, and primary care patients is significantly increasing [4,5]. Therefore, it becomes imperative to explore new formulation strategies in order to improve the efficacy of FA, an antibiotic that is still gaining clinical interest in new markets where it is not registered, such as the US market [6,7]. Several nanotechnology-based formulations were developed for topical skin applications such as liposomes, niosomes, nanocapsules, solid lipid nanoparticles, nanoemulsions, and others [8,9,10,11,12]. These nano-formulations were reported to offer many advantages over conventional topical formulations such as increased drug water-solubility, higher retention of actives in the skin and controlled drug release leading to reduction in the frequency of topical application [13,14,15]. However, most of the above-mentioned nano-formulations suffer from low entrapment efficiency of encapsulated drugs, limited permeability, chemical problems associated with degradation by hydrolysis and/or oxidation and local irritation from the excipients used in these formulations, especially high amounts of surfactants. For these reasons, few nano-formulations are reported to cross the skin barrier effectively, such as dendritic nanoparticles [16,17].

Unlike nanocarriers, for which the nanoparticle itself has to permeate a biological membrane to deliver the loaded drug, nanocrystals (NC) simply diffuse via the formation of a supersaturated solution, which makes them a very promising delivery tool. Although the nanocrystal technology proved to be very successful in the cosmetic field, very few studies have been carried out to widen such application in the pharmaceutical sector. Dermal application of NC of actives with limited water-solubility is also attractive due to their ease of preparation, maximum drug loading capacity (100%), stability, deeper penetration into the skin layers, low incidence of side effects due to exclusion of excipients, reproducibility, and cost of manufacturing [18,19,20].

Studies addressing FA delivery through the dermal route using nanocarriers are many [21,22,23,24]; however, those addressing nanocrystals (NC) are rare, and few of these have linked the optimization of the formulation to the dermal delivery of the active [25]. Passive diffusion is the most common way of drug penetration through the skin upon application of a dermal formulation [26]. NC achieve a higher concentration of free drug in the aqueous phase of the dermal formulation and/or the skin environment, thus resulting in higher diffusion rates as a result of the increase in the concentration gradient (ΔC) over the stratum corneum (SC) when compared to conventional preparations. Also, NC are characterized by increased adhesiveness to surfaces due to the large area of contact with the skin, which makes them superior to micronized powders [27].

The aim of this work is to design and optimize a fast-dissolving drug depot in the water phase of the skin in the form of FA nanocrystals (FA-NC) as a novel approach to treat skin infections. The optimized FA-NC formulation, selected by a full factorial design, will be then subject to lyophilization to enhance its physical stability and allow its easy incorporation into a dermal product. The physico-chemical characteristics of the developed FA-NC before and after lyophilization will be evaluated using different in-vitro, ex-vivo, and microbiological studies. Moreover, the topical anti-bacterial activity and the passive penetration-enhancing effect of a dermal cream formulation containing the developed FA-NC will be tested using a rat excision wound infection model, and results will be compared to marketed FA cream.

## 2. Materials and Methods

### 2.1. Materials

Fusidic acid (FA) was received as a gift from EVA Pharmaceuticals (Giza, Egypt). Pluronics F68 (PF-68; triblock copolymer of polyoxyethylene-polyoxypropylene-polyoxyethylene with an average Mw of 8500 Da), polyvinyl alcohol 4-88 (PVA 4-88; [–CH_2_CHOH–]*_n_* with average Mw 31,000 Da), polyvinylpyrrolidone K30 (PVP-K30; (C_6_H_9_NO)*_n_* with an average Mw of 40,000 Da) were purchased from Sigma Chemical Co. (St Louis, MO, USA). HPLC grade acetonitrile, methanol, and *o*-phosphoric acid were purchased from Prolabo (Paris, France). Mannitol was purchased from Al-Nasr Chemical Co. (Cairo, Egypt). All other chemicals were of analytical grade. Fucidin cream (20 mg/g, Minapharm Pharmaceutical & Chemical Industries, 10th of Ramadan, Egypt, under license of Leo Pharma, Ballerup, Denmark) was used in this study as the reference product.

### 2.2. Preparation of FA-NC

Fusidic acid (FA) nanocrystals (NC) were prepared using a modified bottom-up technique [28]. Exactly 25 mg of FA were dissolved in 2.5 mL acetone and the resulting solution was injected, using syringe gauge 27G, into 25 mL of well-homogenized aqueous solutions containing 50 mg or 100 mg of different nonionic skin-friendly stabilizers such as PVA 4-88, PF-68 and PVP-K30 to result in a drug to stabilizer weight ratio of either 1:2 or 1:4. The resulting mixtures were homogenized at 13,500 rpm using a high-speed homogenizer (Stuart homogenizer SHM1, Staffordshire, UK)) for different homogenization times. FA-NC were precipitated immediately from the anti-solvent solutions. The produced nanosuspensions were further stirred for 1 h for complete evaporation of acetone. Finally, the nanosuspensions were centrifuged at 15,000 rpm for 60 min at 4 °C to separate the formed FA-NC. The separated FA-NC were collected, washed with distilled water, and centrifuged again at the same conditions. The prepared FA-NC were dispersed in their corresponding stabilizer solution used during their preparation to prevent aggregation during storage.

### 2.3. Response Surface Planning of FA-NC Formulations

A 3^2^ × 2^1^ full factorial experimental design was employed to investigate the influence of the process and formulation variables on FA-NC characteristics using Minitab Software, Version 18 (Minitab, LLC., PA, USA). Three numeric factors were set for the experimental design: (1) the type of stabilizer at three levels (X1: PVA 4-88, PF-68 or PVP-K30), (2) drug to stabilizer (D/S) ratio (*w*/*w*) at two levels (X_2_: 1:2 or 1:4) and (3) the time of homogenization at three levels (X_3_: 2 min, 5 min or 10 min). Based on that, full factorial design was found to be suitable for planning the proposed formulations in the form of 18 runs (18 formulations). On the other hand, two responses have been adopted to be tracked for the optimization of the studied factors: (1) particle size (Y_1_: PS) and (2) span value (Y_2_: SV). The composition and characteristics of the prepared formulations (F1→18) are shown in Table 1. All 18 formulations were performed in a duplicate randomized way to satisfy the statistical requirements.

### 2.4. Selection of the Optimized Formulation

The overall desirability value was chosen as the differentiating parameter to compare the 18 formulations [29]. The values of the dependent variables were optimized with optimization criterion set at the smallest PS and lowest SV to yield the system with the highest overall desirability factor. Following selection, the optimized formulation was subjected to lyophilization and further characterization.

### 2.5. Preparation of Freeze-Dried FA-NC

The optimized selected FA-NC formulation (FA-NC-F12) was mixed with 2% mannitol as cryoprotectant and the mixture was frozen at −80 °C for 12 h using an Ultracold Revco Freezer (Thermo Scientific™). The frozen suspensions were then lyophilized in a freeze-drier (Flexi-Dry TM MP Freeze Dryer, SP Scientific, Stone Ridge, NY, USA) at −90 °C and 380 mT for 48 h to yield dry powders. The vials containing the lyophilizates were sealed immediately after removal from the freeze-drier, wrapped in aluminum foil, and stored at room temperature in a desiccator until further used. The optimized lyophilized FA-NC is coded as FA-NC-F12/L.

### 2.6. Micromeritics of FA-NC

#### 2.6.1. Particle Size and Span Value Measurement

Laser diffraction was used to determine the PS and size distribution profile of the prepared FA-NC suspensions (Master seizer Hydro MU 2000, Malvern MU instruments, UK). The d (0.5) was used to assess the PS. The SV was used to assess polydispersity (index from 0–9). Small span values are indicative of narrow particle size distribution. Span values were calculated from Equation (1):

(1) $$Polydispersity\;(span\;value)=\frac{d(0.9)-d(0.1)}{d(0.5)}$$

where *d*(0.9), *d*(0.5) and *d*(0.1) correspond to PS above 90%, 50%, and 10% of the sample, respectively.

#### 2.6.2. Determination of Zeta Potential

In addition to PS and SV measurements, the zeta potential (ZP) of FA-NC was measured by photon correlation spectroscopy using a Zetasizer Nano ZS-90 Instrument (Malvern Instruments, Malvern, UK). Prior to analysis, FA-NC suspensions were suitably diluted with water. Measurements were performed in triplicate using 90° light scattering angle at 25 °C. The mean value ± SD of three replicates was calculated.

### 2.7. Physico-Chemical Characterization of Optimized FA-NC

#### 2.7.1. Determination of Saturated Solubility

The saturated solubility of FA coarse powder, FA-NC-F12, FA-NC-F12/L and FA coarse powder in presence of PVA 4-88 was determined by adding an excess amount of powders in vials containing 10 mL deionized water. The vials were immersed in a thermostatically controlled shaking water bath (Precision Scientific Inc., Chicago, IL, USA) at 37 °C until equilibrium is reached (48 h). Samples were filtered using a 0.1 µm membrane filter (Whatman Inc., Clifton, NJ, USA) and assayed for FA concentration using HPLC [30]. All experiments were conducted in triplicates and the test results are presented as mean value of three determinations ± SD.

#### 2.7.2. In-Vitro Dissolution Studies

The dissolution profiles of FA coarse powder, FA-NC-F12, and FA-NC-F12/L were determined in a dissolution tester (SR8-PLUS; Hanson Research Corporation, Chatsworth, CA, USA) following the USP paddle method. All tests were conducted in 100 mL phosphate buffer at pH = 7.4. The dissolution medium was maintained at 37 °C with a paddle rotation speed of 50 rpm. The amount of drug used was equivalent to 10 mg. At specified time intervals (2, 4, 6, 8, 10, 15, 20, 30, 45, and 60 min), 3 mL of the dissolution media were withdrawn, and replaced with an equal volume of fresh medium to maintain a constant total volume. Samples were filtered and assayed for drug content by HPLC [30] after appropriate dilution. Cumulative amount of drug dissolved was calculated using calibration equation. Dissolution tests were performed in three vessels per formulation (*n* = 3).

#### 2.7.3. Differential Scanning Calorimetry (DSC) Studies

The DSC thermograms of coarse FA, PVA 4-88, mannitol, physical mixture (PM) of FA with PVA, PM of FA with mannitol and FA-NC-F12/L were determined using differential scanning calorimetry (DSC) technique to characterize the degree of crystallinity of FA in the prepared FA-NC. Samples weighing approximately 5 mg were hermetically sealed in aluminum pans and analyzed using DSC 822e (Mettler-Toledo International Inc., Columbus, OH, USA). The samples were heated in an atmosphere of nitrogen at a constant heating rate of 10 °C/min in the range of 25–300 °C.

#### 2.7.4. Powder X-ray Diffraction (XRD)

XRD experiments were performed in X-ray diffractometer (MD-10 mini diffractometer, MTI Corporation, Richmond, CA, USA) using Cu K 2α rays (λ = 1.54056 Å) with a voltage of 25 kV and a current of 30 mA, in a flat plate θ/2θ geometry, over the 2θ ranges 14°–75°. XRD patterns of coarse FA, PVA 4-88, PM of FA with PVA, and FA-NC-F12/L were obtained.

### 2.8. Morphological Characterization

The morphology of FA-NC-F12 and FA-NC-F12/L suspensions was envisioned via transmission electron microscopy (TEM). One drop of the sample was loaded on a copper-gold carbon grid and left to dry in the air. The grid was placed in the vacuum chamber of the electron microscope (JEOL-2100, Jeol Ltd., Tokyo, Japan) and images were captured using different magnifications.

### 2.9. Short-Term Stability Studies

The short-term stability of FA-NC-F12 suspensions and FA-NC-F12/L lyophilizates was determined. The study was carried out at room temperature and 4 °C for 30 days. The vials containing FA-NC were sealed, wrapped in aluminum foil and subdivided into two groups. One group was stored in refrigerator at 4 °C and the other group was stored at room temperature (25 °C) for 30 days. At the predetermined time intervals, samples were taken and subjected to PS analysis and SV determination as described above. The change in appearance (presence of aggregates), PS, and SV were recorded and compared to results obtained from freshly prepared FA-NC.

### 2.10. Skin Permeation Studies

#### 2.10.1. Ex-Vivo Permeation

Newly born albino rat skin was used for permeation studies. Rat skin was reported to carry structural similarities and permeation kinetics comparable to human skin [31]. The excised rat skin was placed in phosphate buffer saline (PBS) at pH 7.4 and was immediately frozen at −80 °C until used. The rate and extent of FA-NC-F12 and FA-NC-F12/L permeation through excised rat skin were investigated and compared to coarse FA as a control. Pieces of rat skin with an approximate surface area of 4.9 cm^2^ were carefully fixed at one end of the specially designed glass cylinders with 2.5 cm internal diameter representing the donor compartment. The stratum corneum (SC) was facing the donor compartment. The other end of the glass cylinders was fitted into the shafts of USP type I dissolution apparatus (SR8-PLUS; Hanson Research Corporation, Chatsworth, CA, USA). FA-NC suspensions equivalent to 10 mg FA, were placed in the donor compartment. The rotation speed was set at 50 rpm and the temperature at 32 °C. 250 mL PBS was utilized as the receptor medium (receptor compartment). At specified time intervals (1, 2, 3, 4, 6, 8, 12, and 24 h) 0.5 mL samples from the receptor medium were withdrawn and immediately replaced with the same volume of buffer solution to maintain a constant volume. Samples were filtered and permeated drug was analyzed by HPLC [30]. The cumulative amount of drug permeated per unit area of skin (μg/cm^2^) into the receptor medium was plotted vs. time. All experiments were carried out in triplicates and the mean values (±SD) were recorded.

#### 2.10.2. Determination of Dermal Penetration (Retention Studies)

At the end of the ex-vivo permeation study, the penetration of FA from FA-NC-F12, FA-NC-F12/L, and coarse FA into the epidermis and dermis was assessed using the tape stripping method as a well-established method to calculate the total amount of penetrated active and the penetration depth [32,33]. The skin was removed from the glass cylinders and after an incubation time of 1 h at 32 °C in PBS, the skin surface was gently washed (3 times) with 1 mL of distilled water then dried using filter paper. The SC was removed by stripping using successively 10 adhesive tapes (Tesa AG, Hamburg, Germany). The adhesive tapes were firmly pressed onto the skin surface and rapidly pulled off with one fluent stroke. Tissues obtained following removal of the SC were taken as the epidermis and the dermis. The first tape was not considered because it represents the drug that did not penetrate the skin. The rest of the tape strips and tissues obtained after stripping the SC were cut into pieces and placed separately into two flasks containing methanol. The flasks were then sonicated for 5 min to extract the drug. The tape strips and tissue suspensions were filtered out and the clear alcoholic solution in each flask was assayed for drug content using HPLC [30]. The cumulative amount of drug retained in the SC and the tissue was calculated as a function of the skin surface area (μg/cm^2^) and compared to the cumulative amount of drug permeated into the receptor compartment (RC).

### 2.11. Incorporation of FA-NC in Topical Formulation

Although the application of FA-NC suspension to the skin is expected to increase the rate of penetration of FA into the skin [34]; yet, from a translational point of view, the developed FA-NC should be incorporated into a dermal product such as cream, ointment, or gel to assess its antibacterial activity. Creams were reported to be more effective vehicles for the incorporation of NC compared to other vehicles such as oleogels and hydrogels [35]. FA-NC-F12 and FA-NC-F12/L were admixed with the water phase of a cream as a vehicle to form a topical product for further studies. The cream was prepared to contain 2% FA (*w*/*w*), 4.1% (*w*/*w*) liquid paraffin, 47% (*w*/*w*) white soft paraffin ointment, 10% (*w*/*w*) propylene glycol, and 36.9% (*w*/*w*) water containing 0.21% citric acid. Fucidin cream (2% *w*/*w*) was used as the control formulation.

#### 2.11.1. In-Vitro Release Studies

The in-vitro release of FA from the three topical formulations, FA-NC-F12 cream, FA-NC-F12/L cream, and commercial Fucidin cream (2% *w*/*w*, Minapharm, Egypt), was assessed using United States Pharmacopeia (USP) V Dissolution Apparatus (SR8-PLUS; Hanson Research Corporation, CA, USA) with slight modification. Exactly 0.5 g of FA cream was spread over the surface of a watch glass (6 cm in diameter) and then covered with a cellulose nitrate membrane having a pore size of 0.45 μm. A waterproof plaster, fixed with equally spaced plastic clips, held the watch glass and the membrane together. This assembly was placed at the bottom of the dissolution vessel containing 250 mL phosphate buffer (pH 7.4). The rotation speed was set at 50 rpm and the temperature at 32 °C. At specified time intervals (5, 10, 15, 30, 45, 60, 120, and 180 min), 3 mL of the dissolution media were withdrawn, and replaced with equal volumes of fresh media to maintain a constant total volume. Samples were filtered, diluted and assayed for drug content by HPLC [30]. A cumulative amount of drug released from the three tested creams was calculated using calibration equation and the percentage of mean cumulative drug released was plotted against time. The in-vitro release tests were performed in three vessels per formulation (*n* = 3).

#### 2.11.2. Microbiological Studies

The antibacterial activity of FA-NC-F12 and FA-NC-F12/L creams against *S. aureus* (ATCC 29737) and *S. epidermidis* (ATCC 12228) was assessed and compared to Fucidin cream using different microbiological studies. Bacterial susceptibility to FA from the three cream formulations was evaluated using the agar diffusion method according to Clinical and Laboratory Standards Institute guidelines of the CLSI [36] by measuring the diameter of the clear inhibition zone in mm. While the minimal inhibitory concentration (MIC) were carried out by microdilution broth assay according to the guidelines of the CLSI [37]. MIC was defined as the lowest concentration that inhibits visible growth (shows no color) compared to control wells (without antimicrobial agent), as indicated by the triphenyl tetrazolium chloride (TTC) staining. From all wells that showed no visible signs of growth (MIC and higher concentrations), loopfuls were inoculated onto Mueller Hinton agar plates by streak plate method [38]. The least concentration that did not show any sign of growth of the tested organisms was considered as the minimal bactericidal concentration (MBC) of the tested formulation against the tested bacterial species. All tests were performed in triplicates and the results were provided as mean values ± SD.

### 2.12. In-Vivo Antibacterial Activity

#### 2.12.1. Animals

The topical antibacterial activity of the developed FA-NC-F12/L incorporated into a 2% (*w*/*w*) cream formulation was evaluated using a rat excision wound infection model. The study protocol was approved by The Institutional Animal Ethics Committee (IAEC) at the National Organization of Drug Control and Research (NODCAR/I/27/19) on 25 July 2019. Twenty-four male albino rats, weighing 0.21–0.25 kg, were used throughout the study. The animals were brought from the animal house of NODCAR and housed in plastic cages at the Animal Care Facility under controlled conditions of temperature (25 °C) and humidity (65%) with a 12 h on/off light cycle. The animals were fed a standard rat diet with free access to water throughout the entire period of the study.

#### 2.12.2. Induction of Infected Wounds

Rats were anaesthetized with (300 mg/kg) chloral hydrate via intraperitoneal injection. The dorsal surface of each rat was shaved with an electrical animal clipper and then cleaned with 70% ethanol. Excision wounds were made by cutting out a predetermined dorsal area (approximately 2.2 cm diameter) of the shaved skin using toothed forceps and pointed scissors. The entire wound was left open [39]. Excision wounds were infected by topical application of 15 µL of *S. aureus* ATCC29737 suspension (containing 5000 CFU/mL) to localize the bacteria in the dermis without causing systemic infection.

#### 2.12.3. Drug Administration and Dosing

Following infection of the wounds, the animals were randomly divided into four treatment groups of 6 rats each (*n* = 6). The four groups were as follows: Group 1: rats were treated with normal saline, Group 2: rats were treated with plain cream (no drug), Group 3: rats were treated with 2% Fucidin cream, and Group 4: rats were treated with 2% FA-NC-12/L cream.

After 24 h of infection, 0.5 g of the corresponding cream was applied topically on the infected wound site of the animals in Groups 2, 3, and 4. For Group 1, 0.5 mL of sterile normal saline was applied to the wound area. The four treatments were applied once a day for 10 days.

#### 2.12.4. Wound Healing Assessment

The rate of wound contraction was measured for each rat every other day for 10 days. The wound area was assessed graphically using a transparency paper and a marker. The wound contraction was expressed as the % reduction of the original wound size [40]. The percentage wound contraction was calculated from Equation (2) as follows:

(2) $$\%\;Wound\;contraction=\frac{wound\;area\;on\;day\;0-wound\;area\;on\;day\;n}{wound\;area\;on\;day\;0}\times 100$$

The mean (±SD) % wound contraction of the six rats in each treatment group was calculated.

#### 2.12.5. Bacterial Load

The number of viable bacteria recovered from the infected open wounds at different time intervals was calculated using Levine’s swab culture technique [41]. The end of a sterile cotton swab was twirled on approximately 1 cm^2^ area of the open wound for 5 s with sufficient pressure to cause minimal bleeding of the underlying tissue. Swabs were then placed in 1 mL sterile water for injection and vortexed to release the microorganisms. The resulting fluid was finally serially diluted, plated on nutrient agar, and incubated at 37 °C for 24 h after which the number of CFU/swab was counted for each treatment group [42].

#### 2.12.6. Macroscopic Examination

Photographic images were also taken every other day using a digital camera (Sony, Tokyo, Japan) to monitor the healing progress of the infected wounds in the different treatment groups.

#### 2.12.7. Histological Examination

After the 10-day period of treatment, rats from each treatment group were euthanized with halothane and the wounded area of the skin was harvested for histological assessment. Sections of the wounds were fixed in 10% formalin saline for 24 h, embedded in paraffin, and then sectioned using a microtome. The sections were collected on glass slides, deparaffinized, and stained with hematoxylin-eosin stain for examination using light electric microscope (Olympus BX-51, Tokyo, Japan) [43].

### 2.13. HPLC Analysis

An isocratic reversed phase validated HPLC method [30] was used for the detection and quantification of FA using an Agilent 1260 infinity LC system (Agilent Technologies, Santa Clara, CA, USA) equipped with quaternary pump, an auto-sampler unit, and a UV detector. The stationary phase was Kromasil C18 (150 mm × 4.6 mm) column packed with a 5-μm-size adsorbent. A mixture of acetonitrile/methanol/0.05 M phosphoric acid (50:10:40 *v*/*v*/*v*) with a pH adjusted to 2.9 was used as the mobile phase at a flow rate of 2 mL/min. The detector was set at 235 nm, and the injection volume was 100 μL. The regression equation for the calibration curve was *y* = 26617*x* − 14.87 and the assay was linear (*r*^2^ = 0.998) in the concentration range of 125 μg/mL–5 ng/mL.

### 2.14. Statistical Analysis

All in-vitro measurements were carried out in independent triplicates and values are presented as mean ± SD unless otherwise noted. Statistics were carried out using Minitab 16 (UK). For comparisons between two groups, two-tailed unpaired Student’s t-test was employed. For multiple comparisons, one-way analysis of variance (ANOVA) followed by Bonferroni’s post-hoc test was utilized. A *p*-value of ≤0.05 was considered statistically significant.

## 3. Results and Discussion

### 3.1. Optimization of FA-NC Formulation

**Figure 1:**
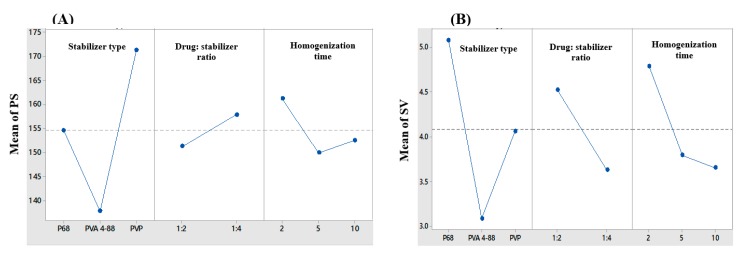
Line plots for the main effects of the type of stabilizer, drug to stabilizer ratio and homogenization time on (**A**) particle size (PS) and (**B**) span value (SV) of the prepared FA-NC.

Nanocrystals (NC) are crystalline drug nanoparticles prepared by different techniques typically to improve the water solubility of poorly soluble drugs [19,44]. Bottom up approaches specifically are reported to produce the smallest PS with lyophilization [45]. Nanocrystals (NC) consist of 100% drug particles surrounded by an adsorbed layer of surfactant or polymer on its surface to prevent aggregation. In this study, NC of FA were prepared successfully using modified bottom-up technique. The effect of the type of the stabilizer, drug to stabilizer ratio, homogenization time and their interactions on PS and SV were statistically analyzed using 3^2^ × 2^1^ full factorial experimental design. Table 1 summarizes the composition and characteristics of the 18 formulations prepared using this design. The 18 formulations possessed a range of PS between 132–194 nm and SV between 1.3 to 6. Upon statistical analysis of the data, the type of the stabilizer used had the most significant effect on both PS and SV (*p* < 0.0001). On the other hand, the drug to stabilizer ratio and the homogenization time had a significant effect on SV (*p* = 0.004 and *p* = 0.006 respectively), but a moderate effect on PS (*p* = 0.026 and *p* = 0.01 respectively). From all 2-way interactions between the independent variables, only the interaction between the type of the stabilizer and the drug to stabilizer ratio was found to have a moderate effect on the PS (*p* = 0.021). The 3-way interactions among the three independent variables also showed a significant effect on PS with no effect on SV (*p* = 0.006 and *p* = 0.208 respectively), thus highlighting the complexity of formulation. Graphical analysis of the main effects of the three operational variables on PS and SV are shown in Figure 1.

The choice of stabilizer is important as it contributes to the overall physical stability of the NC by providing an effective barrier against particle aggregation and crystal growth upon storage [46]. PVA 4-88, a synthetic polymer known to form strong films around the particles and prevent aggregation [47], was the most successful stabilizer in producing the smallest NC with the lowest SV (Figure 1). Statistical analysis revealed that increasing the drug to stabilizer ratio from 1:2 to 1:4 resulted in a slight increase in the PS along with a decrease in the SV. These results might be attributed to a difference in the solubility of FA in the acetone/water mixture in presence of a high concentration of stabilizer compared to a lower one. The solubility of FA in the acetone-water mixture might have increased due to the solubilizing effect of the hydrophilic stabilizer used, leading to a slower rate of drug precipitation and formation of larger crystals. While at a lower concentration, the solubilizing effect of the stabilizer was less pronounced leading to a higher precipitation rate and the formation of smaller crystals. Another explanation could arise from an increase in the viscosity of the aqueous phase with the use of a higher concentration of the stabilizer, which will provide a higher resistance to the diffusion of the drug-solvent phase into the external aqueous phase, thus resulting in larger crystals due to an overall reduction in the counter diffusion rate of the solvents [48]. Increasing the homogenization time from 2 min to 5 min was found to reduce the PS and SV significantly (*p* = 0.01 and *p* = 0.006 respectively). At a high speed of homogenization, longer homogenization times might further aid in reducing the PS due to improved droplet dispersion and reduced droplet coalescence. The improved dispersion and reduced coalescence are expected to decrease the SV as well. However, further increase in the homogenization time to 10 min did not result in any significant reduction in the PS. This could be justified in terms of the percolation theory where discontinuity of a certain property of a system exists above a critical point (percolation threshold).

Optimization results revealed that formulation “F12” is the optimized system when the optimization conditions were set to the smallest PS and the lowest SV with a composite desirability factor of 0.909. A more uniform size distribution of NC was reported to provide a higher physical stability, while a smaller PS was found to improve the passive diffusion of poorly soluble actives and promote their penetration deeper into the skin layers [35]. The selected optimum formulation (F12) was prepared using PVA 4-88 as a stabilizer, a drug to stabilizer ratio of 1:4 and a homogenization time of 10 min. The selected formulation is coded as FA-NC-F12. FA-NC-F12 exhibited a mean PS of 138 nm and SV of 1.3. FA-NC-F12 was further subjected to lyophilization using mannitol as a cryoproctectant to improve the physical stability of the NC, prevent crystal growth and produce dry powder that can incorporated easily into different topical preparations [49]. Also, FA-NC-F12 showed a negative ZP of −12 mV which might contribute to the effective stabilization of the NC through electrostatic repulsion [50]. The optimized freeze-dried formulation is coded as FA-NC-F12/L.

### 3.2. Characterization of Optimized FA-NC

#### 3.2.1. Micromeritics of FA-NC

FA-NC-F12 was freeze-dried successfully and the resulting dry powder had a residual moisture content of less than 1%. The percent process yield was more than 85%. Although freeze-drying is known to improve the physical stability of nanosuspensions, it can, however, result in several physico-chemical changes of the freeze-dried product due to the stresses encountered in the freeze-drying process. There was no significant difference in the PS and SV of FA-NC-F12 following lyophilization, which indicates the resistance of the optimized formulation to the freeze-drying stresses. These results are consistent with our previous results in which it was found that nanoparticles stabilized by polymeric surfactants were more resistant to the freeze-drying stresses compared to non-polymeric surfactants [51]. The characteristics of FA-NC-F12/L are described in Table 2.

#### 3.2.2. Saturation Solubility and Dissolution Studies

**Figure 2:**
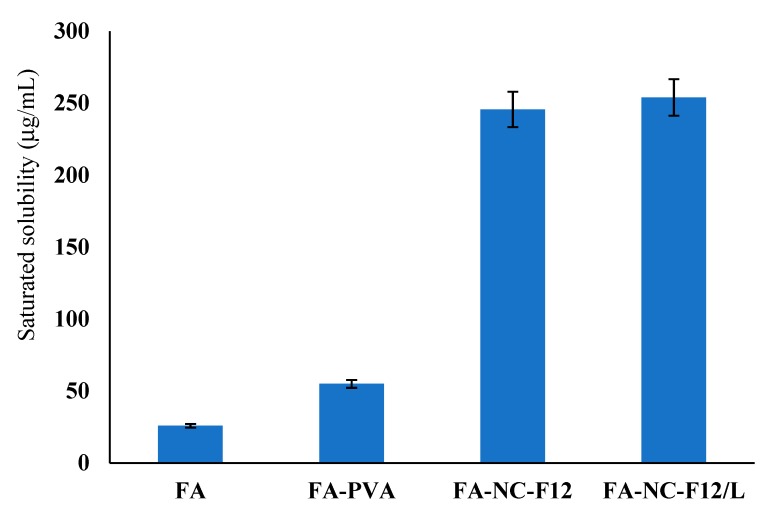
Saturated solubility of FA coarse powder (FA), FA coarse powder dispersed in water containing PVA 4-88 (FA-PVA), FA-NC-F12, and FA-NC-F12/L in pure water at 37 °C.

Limited skin penetration is the major obstacle in dermal drug delivery especially for actives with poor water solubility. The increased saturation solubility of FA-NC is of great importance regarding drug penetration into and permeation through the skin. It is well documented that the saturation solubility of particles less than 1 μm in size increases exponentially with decreasing PS due to increase in dissolution pressure [52,53]. The saturation solubility results are illustrated in Figure 2.

The saturation solubility of coarse FA powder in water was found to be approximately 25.8 ± 1.2 μg/mL while that of FA-NC-F12 was about 245.6 ± 3.2 μg/mL, i.e., almost a 10-fold increase in the saturation solubility compared to coarse FA. The saturation solubility of FA-NC-F12/L was no different compared to the original optimized nanosuspension (FA-NC-F12) amounting to 254.3 ± 9.6 μg/L. These results indicate that the freeze-drying process did not alter the saturation solubility of the original nanosuspension. The saturation solubility of coarse FA in presence of PVA 4-88 (54.88 μg/mL) was almost double that of the raw drug powder. Hydrophilic surfactants are known to increase the water-solubility of poorly soluble drugs due to the adsorption of the polymeric surfactant on the surface of the drug particle; this might lead to better wettability, reduced aggregation, improved dispersibility and faster dissolution of the drug particle once in contact with water.

**Figure 3:**
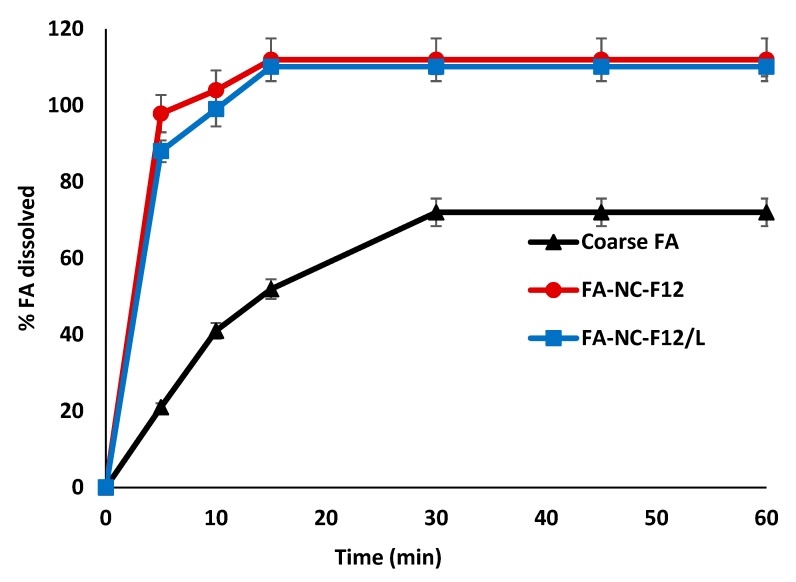
In-vitro dissolution profiles of FA coarse powder, FA-NC-F12 and FA-NC-F12/L in phosphate buffer (pH 7.4) at 37 °C.

Figure 3 shows the dissolution results from the two NC formulations compared to coarse FA powder. In accordance with saturation solubility results, remarkable differences in the rate and extent of FA dissolution were observed from FA-NC compared to FA coarse powder.

The percent drug dissolved in 5 min from FA-NC was almost 100% compared to only 20% from coarse FA powder. The tremendous increase in the dissolution rate of FA-NC is attributed to the significant increase in the saturation solubility and to the large surface area of small drug particles in contact with the dissolution medium, which is in agreement with Noyes-Whitney equation. The dissolution profile of FA from the freeze-dried FA-NC (FA-NC-F12/L) was slightly lower than the original FA-NC-F12 during the first 10 min. A contributing factor might be related to the presence of mannitol in the formulation of FA-NC-F12/L. Mannitol might play a role in hindering the dissolution of FA-NC-F12/L initially. Mannitol being a water-soluble excipient might rapidly dissolve once in contact with water resulting in the formation of a diffusion layer around the NC that could hinder the dissolution of FA and its rapid diffusion into the dissolution medium early in the dissolution process. These results further indicate that the lyophilization process did not alter the physico-chemical properties of the original nanosuspension.

In one study, it was shown that the local concentration of dissolved drug on the site of application and not the concentration in the whole formulation is the determining factor for optimal dermal penetration of actives from NC formulations [54]. In another study, it was reported that the excipients used in the NC formulation have a tremendous influence on the dermal penetration of actives [35].

The saturation solubility and the dissolution results obtained from FA-NC-F12 and FA-NC-F12/L might predict faster drug penetration into the skin layers from applied topical products containing FA-NC thus achieving early effective drug concentration at the site of infection with rapid onset of bacterial growth inhibition compared to conventional products.

#### 3.2.3. DSC and XRD Studies

**Figure 4:**
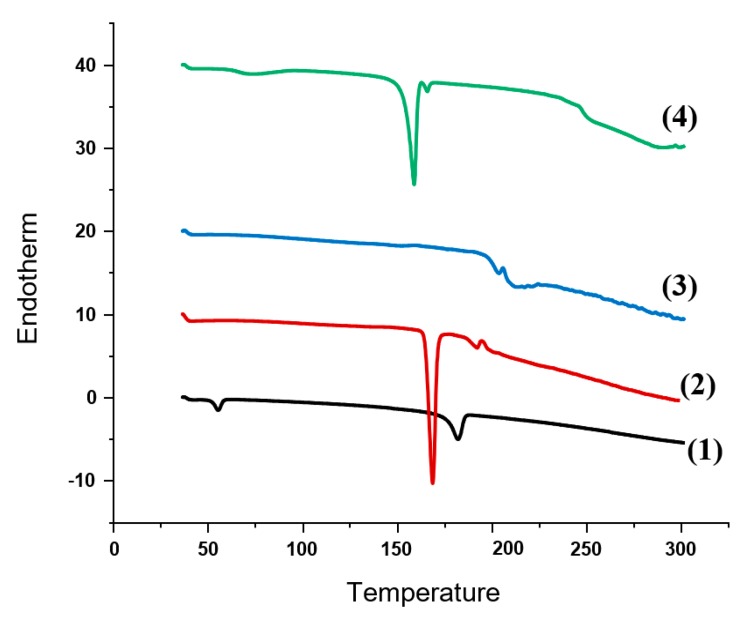
DSC thermograms of FA coarse powder (1); FA: mannitol PM (2); FA: PVA PM (3) and FA-NC-F12/L (4).

In order to identify any change in the physical state and crystallinity of FA upon processing into nanocrystals, DSC and XRD studies were performed. Figure 4 shows the DSC thermograms of FA coarse powder, physical mixture (PM) of FA with PVA 4-88, PM of FA with mannitol and FA-NC-F12/L. The DSC thermogram of FA showed a sharp endothermic peak at nearly 188 °C corresponding to its melting transition point. The thermograms of the PM of FA with PVA and PM of FA with mannitol showed the endotherm indicative of the drug but attenuated and slightly shifted to higher temperature indicating the presence of crystalline drug. The low intensity of the peak could be due to the dilution effect of the drug with the excipients. The DSC thermogram of FA-NC-F12/L showed the small endotherm characteristic of the drug but unlike the PM, the peak was shifted to lower temperature. The observed shift from higher to lower melting point in this case may be due to the processing of FA into nanocrystals, which might lead to a reduction in the melting enthalpy due to the increase in the surface area compared to the heat flow through larger crystals. FA-NC-F12/L thermogram also showed the characteristic strong endothermic peak of mannitol at 168 °C slightly shifted indicating that the lyophilization process did not alter the crystallinity of the drug or mannitol. DSC of PVA and mannitol were also performed but the scans are not included in the figure.

**Figure 5:**
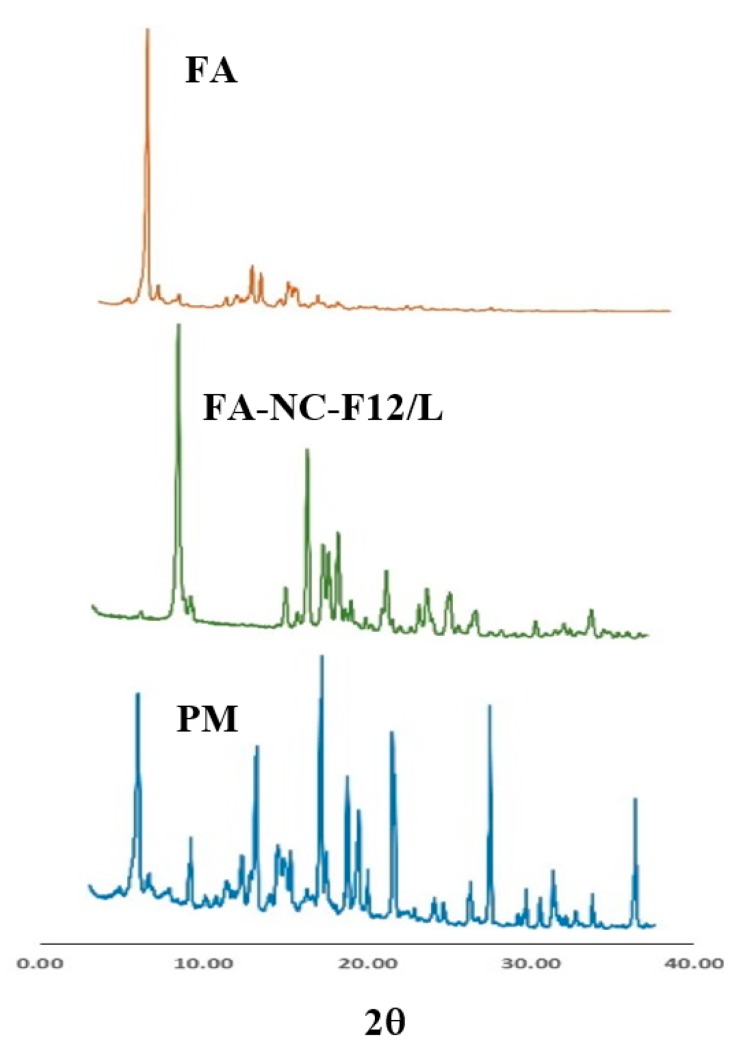
XRD spectra of FA coarse powder, FA-NC-F12/L, and physical mixture (PM) of FA with PVA and mannitol.

To further clarify the solid state of FA in FA-NC formulation, XRD studies were performed as shown in Figure 5. Pure FA exhibited strong and characteristic XRD pattern dominated by intense scattering peaks located at various 2θ positions between 4° and 20°, mainly at 7.2°, 13.7°, 15.9°, 16.4° and 17.9° indicative of the crystalline nature of the drug powder [55]. The diffraction patterns of FA in FA-NC-F12/L retained most of these characteristic peaks with a slight shift to the right indicating that the formulation ingredients and procedure did not change the crystalline nature of the drug. Physical mixture (PM) of FA with PVA and mannitol (1:1:1) exhibited all the characteristics peaks of the three components at the same position.

### 3.3. Morphology and Surface Characteristics Studies

**Figure 6:**
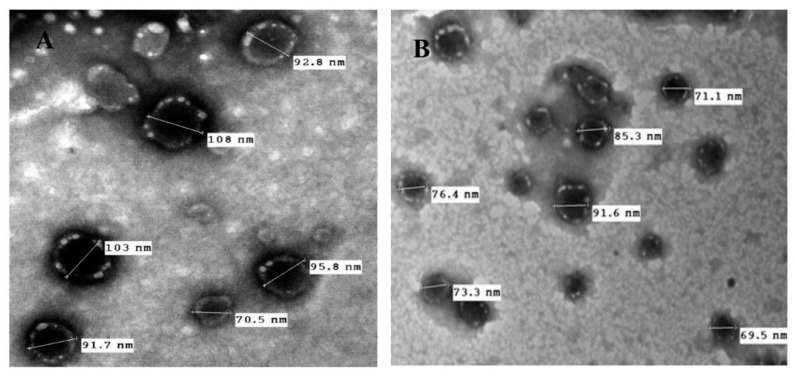
TEM micrographs of (**A**) optimized FA-NC-F12 suspension showing spherical particles with clear distinct thick layer of adsorbed stabilizer molecules around the particle and (**B**) lyophilized FA-NC-F12/L suspension showing no sign of particle aggregation.

TEM micrographs of the original FA-NC-F12 nanosuspension showed spherical nanoparticles with distinct layer of adsorbed stabilizer molecules around the surface of each particle (Figure 6A).

Although PVA is known to form strong adhesive films around particles, this distinct adsorbed layer of PVA could be due to the reported localized gelatinization of PVA on the surface of the particles due to acetone-PVA interaction [56]. TEM micrographs of freeze-dried FA-NC-F12/L showed spherical separate particles embedded in the matrix of mannitol with no sign of particle aggregation further confirming the resistance of the original nanosuspension to the stresses encountered in the freeze-drying process (Figure 6B). These results are also in accordance with the saturation solubility and dissolution results as previously described. The relatively smaller PS obtained from TEM compared to dynamic light scattering technique (DLS) could be due to the reported dehydration effect of TEM imaging [57]. A successful freeze-drying process is imperative to the ease of redispersion of the freeze-dried nanosuspension upon contact with an aqueous phase with no aggregation.

### 3.4. Short-Term Stability Studies

Results from short-term stability studies showed an insignificant increase in the PS of FA-NC-F12 and FA-NC-F12/L upon storage for 30 days in refrigerator. A significant increase in the PS of both FA-NC formulations accompanied by a significant increase in SV was however observed after 30-day storage at room temperature (Table 3). To eliminate possible changes in the PS upon standing, all batches were freshly prepared and used on the same day or stored for no more than 15 days at 4 °C for ex-vivo and in-vivo studies.

### 3.5. Skin Permeation Studies

#### 3.5.1. Ex-Vivo Permeation

**Figure 7:**
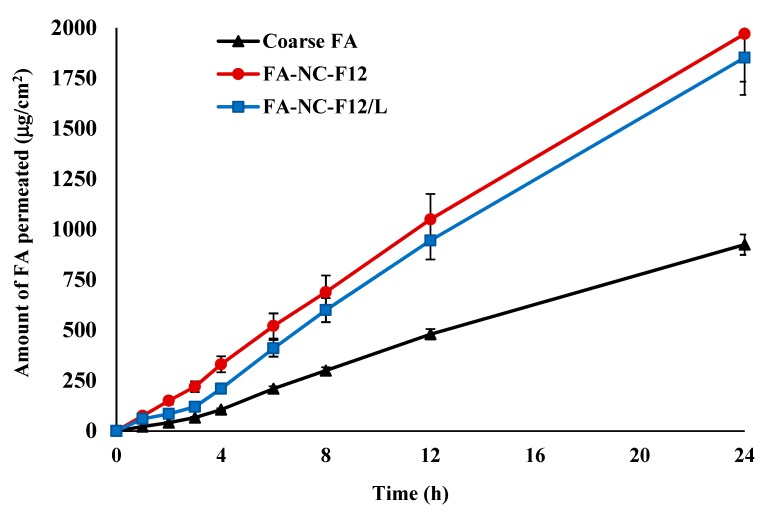
Ex-vivo permeation profiles of FA from FA-NC-F12 and FA-NC-F2/L compared to coarse FA suspension through rat skin in PBS (pH 7.4) at 32 °C.

Ex-Vivo permeation studies were performed to predict the ability of FA-NC to improve FA permeation through the skin layers in-vivo. The permeation profiles of FA-NC-F12 and FA-NC-F12/L through the whole skin compared to coarse FA as a control are shown in Figure 7.

The rate and extent of FA permeation from FA-NC-F12 at all time points and up to 24 h were slightly higher compared to FA-NC-F12/L, but not statistically significantly different. The rate and extent of FA permeation from FA-NC-F12 and FA-NC-F12/L compared to coarse FA on the other hand were statistically significantly higher especially after the first 2 h (*p* > 0.05). The total amount of drug permeated at 24 h was calculated to be more than 112% and 101% higher from FA-NC-F12 and FA-NC-F2/L respectively compared to coarse FA. As skin permeation is concentration dependent, these results are in agreement with saturation solubility and dissolution results indicating that the rapidly dissolving FA-NC from both formulations act as a depot in the water phase of the skin that rapidly replace FA molecules moving out the water phase to penetrate the skin. The slight reduction in the permeation of FA-NC-F12/L compared to FA-NC-F12 might again be due to mannitol used in the lyophilized NC, which might play a role in hampering the penetration of the drug to the skin at the beginning.

#### 3.5.2. Retention Studies

**Figure 8:**
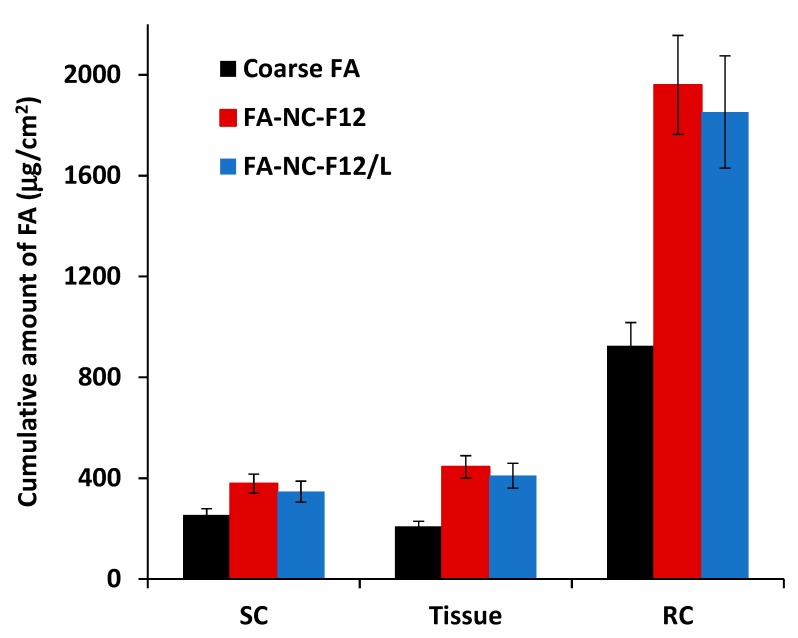
Cumulative amount of FA retained and permeated through rat skin from optimized FA-NC-F12, FA-NC-F12/L or coarse FA in PBS (pH 7.4) in 24 h. SC, stratum corneum; Tissue, epidermis + dermis; RC, receiver compartment.

The amount of drug accumulated per unit area in the different layers of the skin from FA-NC-F12, FA-NC-F12/L and coarse FA were evaluated and compared to the amount of drug permeated per unit area into the receiver compartment (RC) as depicted in Figure 8. Following tape stripping and separation of SC from the rest of the skin (epidermis and the dermis), chemical analysis of the drug showed that the amount of FA accumulated in the SC and tissue (epidermis + dermis) compartment from FA-NC-F12 and FA-NC-F12/L were not statistically significantly different (*p* > 0.05). However statistically higher amount of FA was distinctly detected in the SC and the tissue layers from both FA-NC formulations compared to coarse FA. For instance, the amount of FA retained in the tissue compartment from FA-NC-F12, FA-NC-F12/L, and coarse FA was determined to be 446.8 ± 9.5 μg/cm^2^, 410.1 ± 12.2 μg/cm^2^ and 208.4 ± 8.3 μg/cm^2^ respectively representing almost a 2-fold increase for the amount of FA retained in the skin tissue from the FA-NC formulations.

These results could be attributed to the tremendous increase in the saturation solubility of FA-NC, which might result in higher concentration gradient across the SC with the subsequent increase in the diffusive flux into the skin. Another contributing factor to the increased dermal penetration of the NC might be due to the increase in the total surface area of contact between the NC and the skin through distribution in the hair follicles. It has been reported that particles below 1 μm preferentially accumulate in the hair follicles [58] and it is acknowledged that the smaller the particle is the deeper the localization in the hair follicles will be [59,60]. Also, FA-NC might possess, as previously reported for nanosized materials, an increased adhesiveness to biological membranes [35], thus resulting in longer retention time at the site of application leading to higher drug percutaneous absorption. From a translational point of view, FA-NC can be tuned in term of PS to either localize drugs into the skin layers to result in a local action with reduced systemic drug delivery and toxicity or can be tailored for transdermal delivery to result in high systemic drug bioavailability due to high down deeper permeation through the entire skin.

Taken together, in-vitro and ex-vivo results FA-NC-F12 and FA-NC-F12/L were incorporated into a topical dermal product and then subjected to further testing using marketed Fucidin cream as a control.

### 3.6. Evaluation of FA-NC in Topical Formulation

From a translational point of view, the developed FA-NC should be incorporated into a dermal product such as cream, ointment or gel to assess its antibacterial activity. Not only creams are reported to be effective vehicles for the incorporation of NC for enhanced skin permeation compared to other vehicles [35] but also vehicles and excipients are reported to greatly affect the penetration of incorporated actives into the skin [35,61]. For these reasons, a cream formulation was chosen to incorporate FA-NC to eliminate the vehicle effect when using marketed Fucidin cream as a control in the following studies. The rheological properties of the prepared FA-NC creams were comparable to those of marketed Fucidin cream to further eliminate the vehicle effect. Both creams showed plastic behavior with shear-thinning properties.

#### 3.6.1. In-Vitro Release Studies

**Figure 9:**
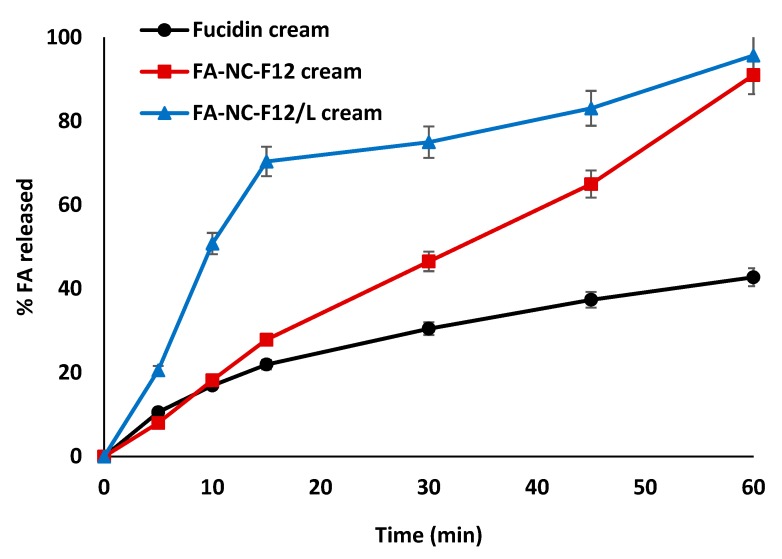
In-vitro release profiles of FA from FA-NC-F12, FA-NC-F12/L and Fucidin creams in PB (pH = 7.4) at 32 °C.

In-vitro release studies were performed to determine the release rate (flux) and percentage (%) cumulative amount of FA permeated through a cellulose membrane from the three tested cream formulations. The in-vitro release profiles of FA from FA-NC-F12, FA-NC-F12/L and Fucidin creams are shown in Figure 9.

Remarkable differences in the rate and extent of FA release from the three cream formulations were observed. Surprisingly, the release of FA from FA-NC-F12/L cream exhibited a significant increase in rate and extent compared to its release from FA-NC-F12 and Fucidin creams especially during the first 15 min (*p* < 0.0001). For instance, the average percentage drug released from FA-NC-F12/L cream after 15 min was 70% compared to only 28% and 22% from FA-NC-F12 and Fucidin creams respectively. These results are not in agreement with previous saturation solubility and dissolution results in which it was found that both FA-NC formulations exhibited similar saturation solubility and dissolution profile. This difference may arise from the setting used in the release studies vs. dissolution studies. In dissolution studies, free FA-NC-F12 and FA-NC-F12/L were in direct contact with the dissolution medium so it is expected that particles with more or less the same size to dissolve at almost the same rate. On the other hand, other factors might play a role in the dissolution of the drug in the water phase of the cream with its subsequent release and diffusion through the cellulose membrane, which make the prediction of the results more complex. A possible explanation might arise from the use of hydrophilic mannitol in the formulation of FA-NC-F12/L. Hydrophilic mannitol might promote the rapid penetration of the release medium by osmosis into the cream thus resulting in the rapid dissolution of the drug in the water phase of the cream and its subsequent fast release followed by its diffusion through the cellulose membrane. The incorporation of a lyophilized product into a cream might as well result in more homogeneous and uniform dispersion of the particles. These results are consistent with the superiority of using a lyophilized product that result in better wettability, reduced aggregation, improved particle dispersibility and faster dissolution when incorporated in dosage forms or once in contact with water [62]. These results further confirm that the release of drugs from dosage forms is greatly affected by the choice of the dosage form and the excipients [35].

The average percentage drug released from FA-NC-F12/L and FA-NC-F12 creams after 60 min was about 95% and 91% respectively compared to 42% from Fucidin cream. The increased saturation solubility of FA-NC is expected to cause high local concentration of dissolved FA in the aqueous phase of the cream compared to the standard marketed formulation. Based on Fick’s law, the amount of dissolved active in the vehicle is a major driving force for the diffusion of the active through, in this case, the cellulose membrane into the release medium. These results also indicate that the cream formulation is a suitable vehicle to incorporate FA-NC especially in the lyophilized form. These findings also suggest that different excipients used in the formulation might lead to different interactions that will promote the release of actives in different ways and/or make the results unpredictable.

#### 3.6.2. Microbiological Studies

Topical use of FA is considered as being one of the best narrow-spectrum antibiotic for treating skin and soft-tissue infections. FA is primarily active against various strains of *staphylococci* including methicillin-resistant and most coagulase-negative *staphylococci*. The comparative microbiological studies, carried out with respect to conventional topical Fucidin cream, revealed the difference in antibacterial activity among the three tested FA cream formulations. Results from microbiological tests are described in Table 4.

FA-NC-F12 and FA-NC-F12/L creams showed more effective antibacterial activity compared to Fucidin cream as indicated by their significantly larger inhibition zones (*p* < 0.05). It is acknowledged that the smaller the PS is, the greater the extent of uptake through a barrier membrane e.g., bacterial cell wall which is usually followed by higher cellular uptake [63]. The antibacterial activity of FA-NC-F12/L and FA-NC-F12 creams against both *S. aureus* and *S. epidermidis* was not statistically significantly different (*p* > 0.05). These results were further confirmed by MIC studies. The observed MIC from FA-NC-F12 and FA-NC-F12/L creams was estimated to be 4 times lower against *S. aureus* and *S. epidermidis* compared to Fucidin cream (*p* < 0.05). On the other hand, MBC values were high from the three formulations and were not statistically different which is in agreement with FA being a bacteriostatic rather than a bactericidal antibiotic.

Based on the results obtained from in-vitro release and microbiological studies, FA-NC-F12/L cream was chosen to be further tested using in-vivo antibacterial studies.

#### 3.6.3. In-Vivo Studies

**Figure 10:**
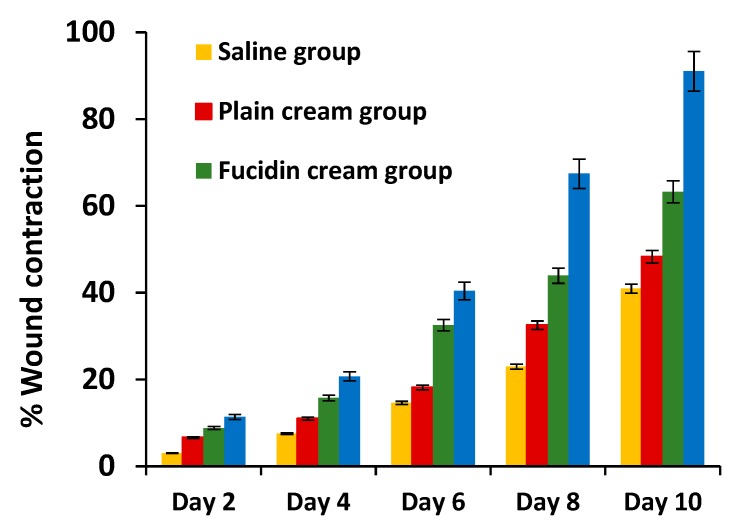
Percentage (% ± SD) wound contraction in the four treatment groups during 10-day treatment with respective formulations.

**Figure 11:**
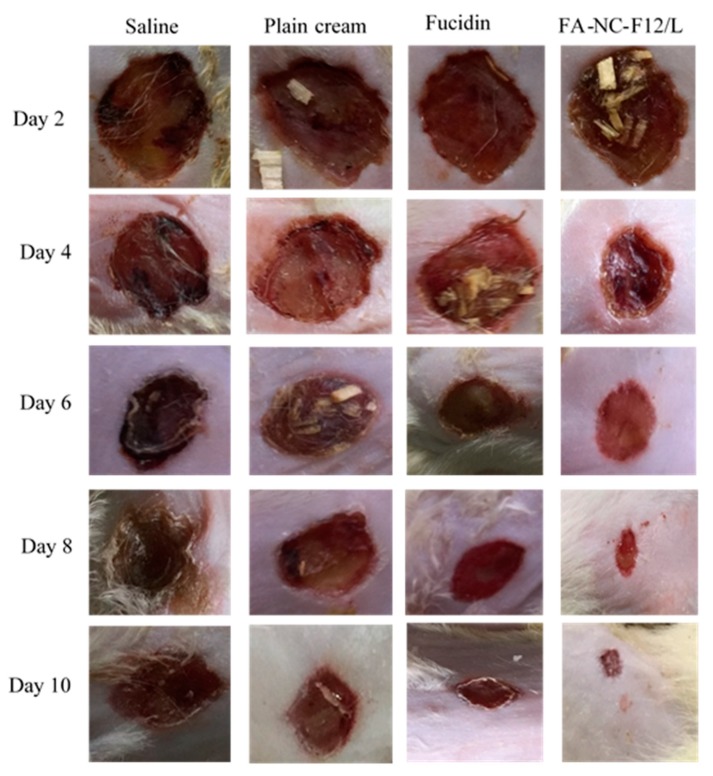
Macroscopic changes in skin wound site in the different groups during the 10-day treatment with respective formulations.

**Figure 12:**
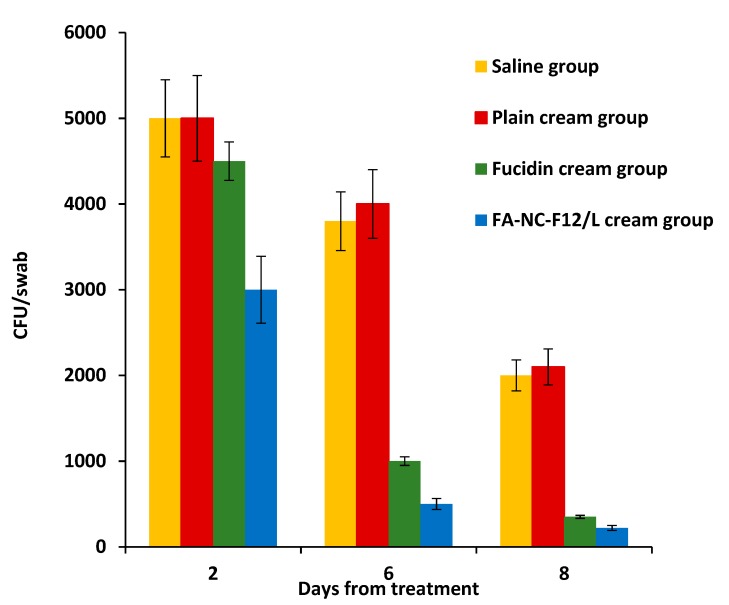
CFU/swab (±SD) collected from infected area of the skin of rats in the four treatment groups at day-2, day-6 and day-8 from treatment with respective formulations (*n* = 6).

**Figure 13:**
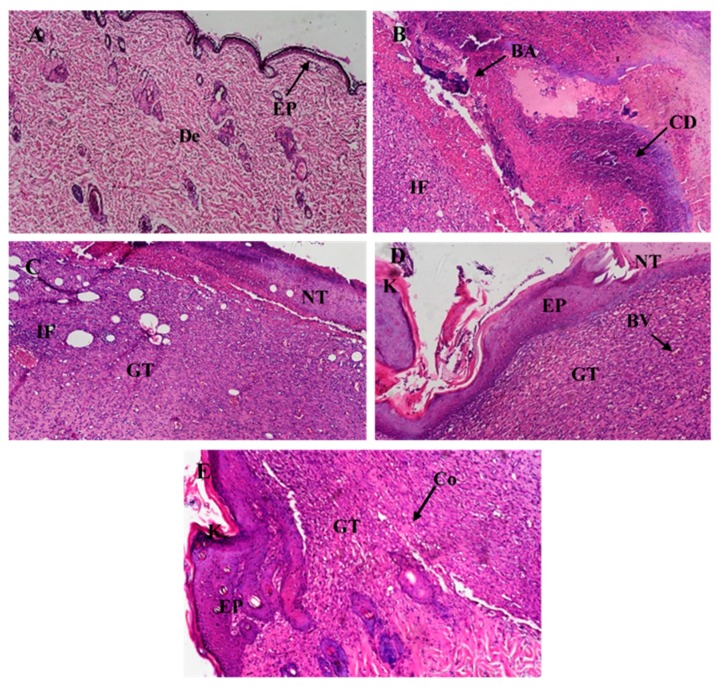
Micrographs of histological sections of healthy skin of normal rat (**A**) and wounded infected skin of rats treated for 10 days with normal saline (**B**), plain cream (**C**), Fucidin cream (**D**) and FA-NC-F12/L cream (**E**). All sections stained with hematoxylin and eosin are displayed at 200× magnification. (EP) epidermal layer, (De) dermal layer, (IF) inflammatory cells infiltration, (GT) granulation tissue, (NT) necrotic tissue, (BA) bacterial aggregate, (HG) hemorrhage, (BV) blood vessel, (CD) cellular debris, (K) keratinization and (Co) collagen.

The results obtained from performing in-vivo antibacterial studies using a rat excision wound infection model are described in Figure 10, Figure 11, Figure 12 and Figure 13. The percentage wound contraction measured every other day during the 10-day treatment with the respective formulations for the four treatment groups is shown in Figure 10.

The percentage wound contraction calculated from day 2 to day 10 from infection was statistically significantly higher in FA-NC-F12/L group compared to Fucidin group (*p* > 0.05). After the 10-day treatment, the mean estimate of the percentage wound contraction in FA-NC-F12/L group (>90%) was determined to be 141%, 184% and 217% relative to the mean estimates calculated for Fucidin, plain cream and saline groups respectively. Figure 11 also shows photographic images of the healing progress of the wounds in the different groups over the 10-day period of treatment.

These images clearly show that FA-NC-F12/L cream enhanced the killing of the bacteria in the infected wound area and therefore exhibited the fastest wound healing ability as indicated by the restoration of almost normal skin appearance compared to the other groups. It was noticed that treatment with plain cream showed an improved wound contraction effect compared to the saline group, which is consistent with the well-known healing properties of many of the excipients used in the cream formulation.

These results were further confirmed by counting the number of CFU/swab in wound swabs collected from the infected rats in each group at days 2, 6, and 8 from treatment as shown in Figure 12.

The one-way ANOVA followed by Bonferroni’s multiple comparison test showed a significantly greater reduction in the number of CFU of *S. aureus* in the group treated with FA-NC-F12/L cream in comparison to the other groups at all tested days. The mean bacterial load estimates (CFU/swab) in Fucidin cream group, at day 2, day 6, and day 8 from treatment, were determined to be about 150%, 200%, and 160% higher relative to the mean bacterial load estimates in FA-NC-F12/L cream group respectively. These results show that the incorporation of FA-NC-F12/L into a cream formulation was more effective in the management of *S. aureus* infections compared to Fucidin cream.

Histological analysis of harvested skin tissue from the wounded area of the skin of the infected animals in the four groups after 10-day treatment with respective formulations was also performed as indicated in Figure 13.

Microscopic examination of normal skin sections revealed normal histological structure of the epidermal layer and the dermal layer (Figure 13A). Examination of rat skin after the induction of an infected wound followed by a 10-day treatment with the respective formulation revealed several histological alterations in the different groups. Animals treated with normal saline presented histopathological characteristics typical of infection, presenting a huge area of necrotic tissue with cellular debris and bacterial aggregates around the wound, along with dermal granulation tissue heavily infiltrated with inflammatory cells (Figure 13B). Animals treated with plain cream also presented histology similar to the group treated with saline (Figure 13C). On the other hand, skin sections of animals treated with Fucidin cream showed beginning of re-epithelization in most wounds extended under the necrotic tissue with scattered keratinization while the rest of the wound gap was covered with necrotic tissue under which granulation tissue was heavily infiltrated with inflammatory cells and blood vessels (Figure 13D). Skin sections of animals treated with FA-NC-F12/L cream presented histological differences compared to Fucidin group. In this group it was possible to observe the presence of an extended re-epithelization layer showing focal separation with near to normal cells while the granulation tissue showed decreased intensity of inflammatory cells with the appearance of small focal areas of collagen deposition in the dermal layer (Figure 13E).

Taken together with results from in-vitro and in-vivo studies, the superior antibacterial activity of FA-NC-F12/L cream reflects not only the increase in the saturation solubility of incorporated FA-NC but also the changes in the physico-chemical properties of the drug molecule. These changes were able to overcome the barrier properties of the skin, facilitate drug permeation deep into the skin, guarantee maximal bacterial exposure in the infected wound and probably localize the drug molecules close to the target receptors present on infective microorganisms thus resulting in increased therapeutic efficacy.

Also, these results demonstrate the suitability of incorporating lyophilized FA-NC into a cream formulation for the effective treatment of *S. aureus* infections. In this cream formulation, FA was able to escape the oily phase of the cream and partition into the hydrophobic-hydrophilic skin structure more effectively than larger FA particles in the marketed cream. This can be explained by the high number of small particles in the NC formulation, which allow the drug to be more evenly distributed over the skin surface resulting in more even distribution of the concentration gradient along the treated skin area [64]. More specifically, incorporating lyophilized FA-NC into a cream improved FA distribution and guarantied maximal bacterial exposure in the infected wound, resulting in increased therapeutic efficacy when compared to coarse FA marketed as Fucidin cream.

Overall, these results demonstrate the great capacity of the FA-NC-F12/L formulation to deliver the antibiotic into the intracellular milieu of the infected skin and enhance its intracellular bacterial killing.

## 4. Conclusions

This study reports the great potential of using a nanocrystal lyophilized formulation of poorly-water soluble FA to improve its saturation solubility, local bioavailability, and efficacy upon topical application to the skin. The incorporation of lyophilized FA-NC into a cream formulation also proved to be very advantageous, resulting in a rapid and complete drug diffusion out of the cream product. Moreover, in-vivo studies revealed the superior antibacterial activity and wound healing ability of FA-NC-F12/L cream compared to the marketed cream. This study highlights the synergistic effect of nanocrystallization and lyophilization when used simultaneously in producing a topical product with superior skin penetration and skin retention, thus allowing the efficient localization of the drug at the site infection. Since the technology for the production scale of manufacture is now available, we think that the developed FA-NC formulation is feasible for easy industrialization, scale-up, and manufacturing. In conclusion, the developed FA nanocrystal formulation is a promising therapeutic alternative to conventional topical FA products in the treatment of skin infections.

## Figures and Tables

**Table 1:** A 3^2^ × 2^1^ full factorial experimental design for the effect of formulation/process variables on the particle size (PS) and span value (SV) of FA-NC.

Formulation Code	Stabilizer Type	D/S ratio	Homogenization Time (min)	PS (nm)	SV
F1	PF-68	1:2	2	155 ± 19	6.0 ± 1.4
F2	PF-68	1:2	5	151 ± 17	5.0 ± 0.3
F3	PF-68	1:2	10	154 ± 16	3.9 ± 0.1
F4	PF-68	1:4	2	165 ± 14	5.6 ± 0.2
F5	PF-68	1:4	5	158 ± 11	4.8 ± 0.7
F6	PF-68	1:4	10	142 ± 27	4.8 ± 1.5
F7	PVA 4-88	1:2	2	140 ± 14	4.5 ± 0.0
F8	PVA 4-88	1:2	5	139 ± 15	2.4 ± 1.7
F9	PVA 4-88	1:2	10	140 ± 21	3.9 ± 0.7
F10	PVA 4-88	1:4	2	137 ± 12	3.8 ± 0.1
F11	PVA 4-88	1:4	5	132 ± 10	2.3 ± 1.6
*F12	PVA 4-88	1:4	10	138 ± 22	1.3 ± 0.0
F13	PVP-K30	1:2	2	188 ± 11	5.3 ± 0.1
F14	PVP-K30	1:2	5	147 ± 12	4.6 ± 0.0
F15	PVP-K30	1:2	10	146 ± 8	4.6 ± 0.2
F16	PVP-K30	1:4	2	181 ± 13	3.2 ± 0.2
F17	PVP-K30	1:4	5	172 ± 11	3.3 ± 0.4
F18	PVP-K30	1:4	10	194 ± 18	3.1 ± 0.1

Data are mean value ± SD (*n* = 3). * The optimized system.

**Table 2:** Composition and characteristics of FA-NC-F12/L.

**Composition (% *w*/*v*)**	
FA	0.1
PVA 4-88	0.2
Mannitol	2.0
**Responses**	
PS (nm)	115.1 ± 13
SV	1.60 ± 0.00
ZP (mV)	−11.6 ± 3.6

Data are mean value ± SD (*n* = 3).

**Table 3:** Particle size (PS) and span value (SV) of FA-NC after 30-day storage at room temperature or 4 °C.

FA-NC	Storage Conditions	PS (nm)	SV
FA-NC-F12	Fresh	138 ± 22	1.3 ± 0.00
	25 °C	662.7 ± 42 *	3.8 ± 0.6 *
	4 °C	147 ± 15	1.4 ± 0.2
FA-NC-F12/L	Fresh	115.1 ± 13	1.6 ± 0.00
	25 °C	454.7 ± 21 *	2.4 ± 0.3 *
	4 °C	121 ± 14	1.7 ± 0.1

Data are mean value ± SD; * *p* < 0.05

**Table 4:** Mean results (±SD) from microbiological tests performed on FA from the three tested cream formulations.

Test	FA-NC-F12	FA-NC-F12/L	Fucidin
**Inhibition zone (mm)**			
*S.aureus*	34.7 ± 1.5 ^a,^*	37.2 ± 1.2 ^a,^*	25.3 ± 0.57
*S. epidermidis*	38.1 ± 1.4 ^a,^*	40.2 ± 1.6 ^a,^*	32.3 ± 0.58
**MIC (μg/mL)**			
*S.aureus*	32 ^a,^*	32 ^a,^*	>128
*S. epidermidis*	16 ^a,^*	16 ^a,^*	64
**MBC (μg/mL)**			
*S.aureus*	>128	>128	>128
*S. epidermidis*	128	128	128

* *p* < 0.05. ^a^ Versus Fucidin (control).
